# Supplementary material for: Identification and Functional Analysis of a Defensin CcDef2 from Coridius chinensis
Source: Int J Mol Sci. 2022 Mar 3;23(5):2789. doi: 10.3390/ijms23052789 (PMC8911331; doi:10.3390/ijms23052789)
Supplement: Supplementary file 1 [file ijms-23-02789-s001.zip › ijms-1600351-supplementary.pdf]

```
                                                M   G   S   S  [H   H   H   H   H   H]  S   S   G   L   V   P ↓ R   G   S   H   M
                                               ATGGGCAGCAGCCATCATCATCATCATCACAGCAGCGGCCTGGTGCCGCGCGGCAGCCATATG
Protein          A  T  C  D  A  L  S  F  Q  S  K  W  I  T  I  N  H  S  A  C  A  I  H  C  I  A  K  G  Y  K
Original 1     GCCACTTGCGACGCACTCAGCTTCCAATCCAAGTGGATCACCATAAACCACTCAGCATGCGCCATCCACTGCATCGCCAAAGGATACAAG
Optimized      GCCACCTGCGATGCCCTGAGTTTTCAGAGCAAATGGATTACCATTAATCATAGTGCATGCGCAATTCATTGCATTGCCAAAGGTTATAAA
Protein         G  G  Q  C  K  N  T  V  C  H  C  R  K  G  G  G  G  S  G  G  G  G  S  G  G  G  G  S  A  T
Original 91    GGTGGTCAATGTAAGAACACTGTCTGTCACTGCAGGAAGGGCGGCGGTGGTAGCGGCGGCGGTGGTAGCGGCGGCGGTGGTAGCGCCACT
Optimized      GGCGGTCAGTGCAAAAATACCGTGTGTCATTGCCGCAAAGGCGGCGGTGGTAGCGGCGGCGGTGGTAGCGGCGGCGGTGGTAGCGCCACC
Protein         C  D  A  L  S  F  Q  S  K  W  I  T  I  N  H  S  A  C  A  I  H  C  I  A  K  G  Y  K  G  G
Original 181   TGCGACGCACTCAGCTTCCAATCCAAGTGGATCACCATAAACCACTCAGCATGCGCCATCCACTGCATCGCCAAAGGATACAAGGGTGGT
Optimized      TGCGATGCCCTGAGTTTTCAGAGCAAATGGATTACCATTAATCATAGTGCATGCGCAATTCATTGCATTGCCAAAGGTTATAAAGGCGGT
Protein         Q  C  K  N  T  V  C  H  C  R  K  G  G  G  G  S  G  G  G  G  S  G  G  G  G  S  A  T  C  D
Original 271   CAATGTAAGAACACTGTCTGTCACTGCAGGAAGGGCGGCGGTGGTAGCGGCGGCGGTGGTAGCGGCGGCGGTGGTAGCGCCACTTGCGAC
Optimized      CAGTGCAAAAATACCGTGTGTCATTGCCGCAAAGGCGGCGGTGGTAGCGGCGGCGGTGGTAGCGGCGGCGGTGGTAGCGCCACCTGCGAT
Protein         A  L  S  F  Q  S  K  W  I  T  I  N  H  S  A  C  A  I  H  C  I  A  K  G  Y  K  G  G  Q  C
Original 361   GCACTCAGCTTCCAATCCAAGTGGATCACCATAAACCACTCAGCATGCGCCATCCACTGCATCGCCAAAGGATACAAGGGTGGTCAATGT
Optimized      GCCCTGAGTTTTCAGAGCAAATGGATTACCATTAATCATAGTGCATGCGCAATTCATTGCATTGCCAAAGGTTATAAAGGCGGTCAGTGC
Protein         K  N  T  V  C  H  C  R  K  *
Original 451   AAGAACACTGTCTGTCACTGCAGGAAGTAG
Optimized      AAAAATACCGTGTGTCATTGCCGCAAATAACTCGAG
```

Figure S1. The optimized sequence of *CcDef2*. The box indicates the 6×His tag. The thrombin site is shaded and the red arrow represents the cleavage site. Mutated nucleotides are shown in red and linkers in blue. Restriction endonuclease sites (*Nde* I and *Xho* I) are indicated using underlines and the asterisk denotes the stop codon.
